# Synthesis, antimalarial, antileishmanial evaluation, and molecular docking study of some 3-aryl-2-styryl substituted-4(3*H*)-quinazolinone derivatives

**DOI:** 10.1186/s13065-022-00903-0

**Published:** 2022-12-02

**Authors:** Girma Worku Seifu, Yihenew Simegniew Birhan, Botros Youssef Beshay, Ariaya Hymete, Adnan Ahmed Bekhit

**Affiliations:** 1 Department of Chemistry, College of Natural and Computational Sciences, Debre Markos University, P.O. Box 269, Debre Markos, Ethiopia; 2 Department of Pharmaceutical Chemistry, College of Pharmacy, Arab Academy for Science, Technology and Maritime Transport, Alexandria, 21913 Egypt; 3 Department of Pharmaceutical Chemistry and Pharmacognosy, School of Pharmacy, Addis Ababa University, P.O. Box 1176, Addis Ababa, Ethiopia; 4 Department of Pharmaceutical Chemistry, Faculty of Pharmacy, Alexandria University, Alexandria, 21215 Egypt; 5 Pharmacy Program, Allied Health Department, College of Health and Sport Sciences, University of Bahrain, Manama, Kingdom of Bahrain

**Keywords:** 2-styryl-4(3*H*)-quinazolinone, Antimalarial activities, Antileishmanial activities, Molecular docking

## Abstract

Quinazolinones are a diverse group of nitrogen-containing heterocyclic compounds with promising antimalarial and antileishmanial activities. Herein, some 3-aryl-2-styryl substituted-4(3*H*)-quinazolinones were synthesized via cyclization, condensation, and hydrolysis reactions. ^1^H NMR, FTIR and elemental microanalysis was used to verify the structures of the synthesized compounds. The in vivo antimalarial and in vitro antileishmanial activities of the target compounds were investigated using mice infected with *Plasmodium berghi* ANKA and *Leishmania donovani* strain, respectively. Among the test compounds,** 8** and **10** showed better antimalarial activities with percent suppression of 70.01 and 74.18, respectively. In addition, (*E*)-2-(4-nitrostyryl)-3-phenylquinazolin-4(3*H*)-one (**6)** showed promising antileishmanial activity (IC_50_ = 0.0212 µg/mL). It is two and 150 times more active than the standard drugs amphotericin B deoxycholate (IC_50_ = 0.0460 µg/mL) and miltefosine (IC _50_ = 3.1911 µg/mL), respectively. Its superior in vitro antileishmanial activity was supported by a molecular docking study conducted in the active site of Lm-PTR1. Overall, the synthesized 3-aryl-2-styryl substituted-4(3*H*)-quinazolinones showed promising antileishmanial and antimalarial activities and are desirable scaffolds for the synthesis of different antileishmanial and antimalarial agents.

**Supplementary Information:**

The online version contains supplementary material available at 10.1186/s13065-022-00903-0.

## Introduction

Malaria and leishmaniasis are the most debilitating tropical infectious diseases caused by the protozoa belonging to the genus *Plasmodium* and *Leishmania*, respectively [1–3]. According to WHO 2021 report, nearly half of the world’s population were at risk of malaria. It accounted for an estimated 241 million cases and 627, 000 deaths worldwide in 2020. Disruptions in malaria services due to the COVID-19 pandemic are partly responsible for the increase in malaria cases by 14 million and deaths by 69,000 as compared to the previous year [4]. Leishmaniasis is prevalent in at least 98 countries with an estimated 350 million people at risk. The estimated global prevalence of all forms of the disease is 12 million, with about 0.9–1.6 million new cases and between 20,000 and 30,000 deaths each year. In 2019, around 0.6–1 million new cases of cutaneous leishmaniasis (CL), and some 50,000–90,000 new cases of visceral leishmaniasis (VL) were reported worldwide [5]. The latter also triggered the loss of 1.4 million disability-adjusted life-years (DALYs) in 2015, representing approximately 6% of all the DALYs instigated by neglected tropical diseases (NTD) [3]. Although there are potent and rapidly acting antimalarial and antileishmanial drugs available in the clinics, their widespread use triggered treatment failures and the emergence of drug-resistant strains [6]. Different reports revealed that artemisinins and artemisinin combination therapies (ACTs), the most effective antimalarial drugs, have already developed resistance in different parts of the world [7–9]. Several reports reiterated that the efficacy of antileishmanial drugs such as pentavalent antimonials (Sb^V^) [10], miltefosine [11, 12], paromomycin [13], and pentamidine [14] is rendered by treatment failure and the emergence of drug-resistant strains. The risk of resistance development for amphotericin B is also apparent [15, 16]. Overall, screening of safe, effective and affordable antimalarial and antileishmanial agents is highly desirable to curb the morbidity and mortality associated with malaria and leishmaniasis.

**Fig. 1:**
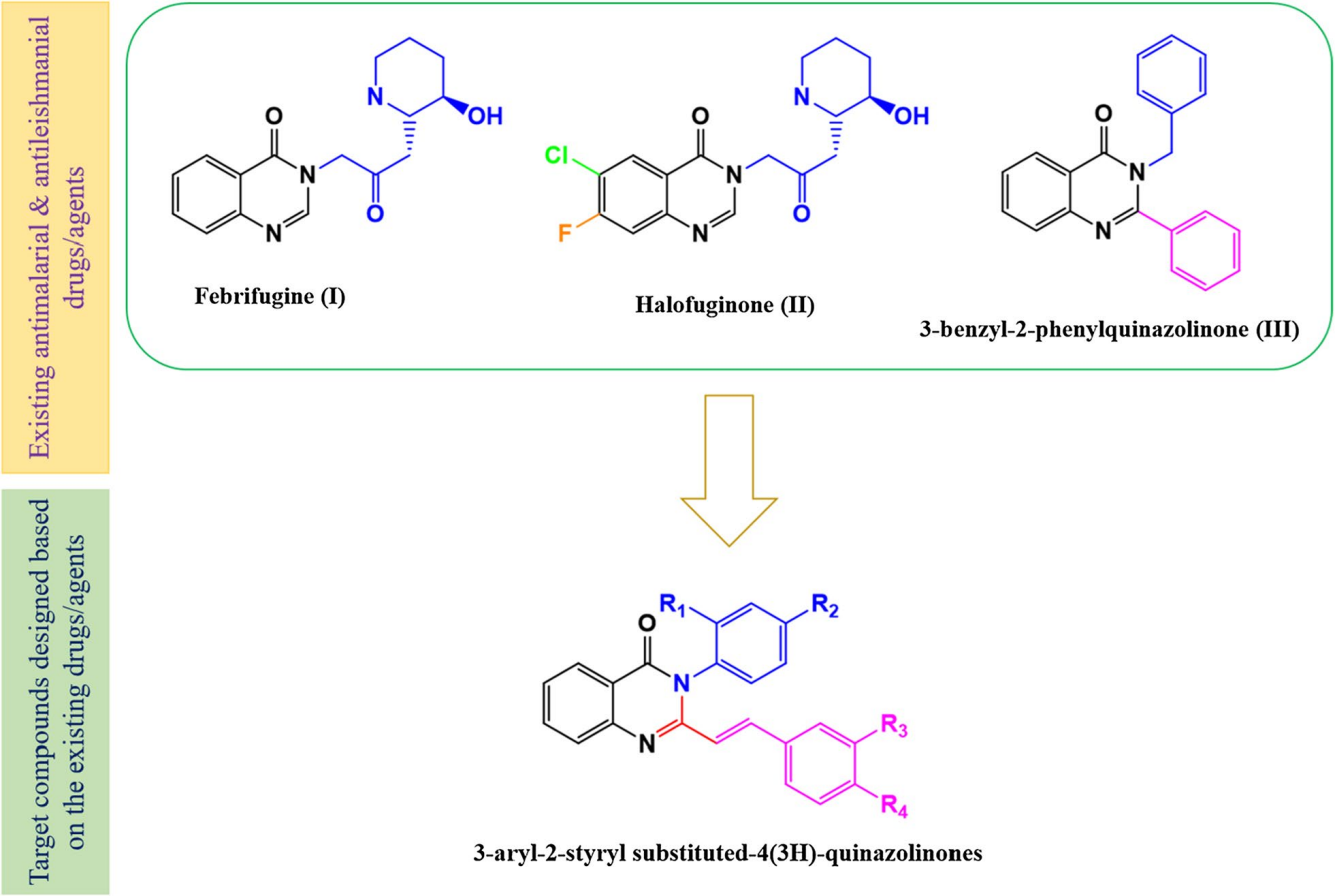
Design rationale of 3-aryl-2-styryl substituted-4(3*H*)-quinazolinone derivatives based on the existing antimalarial and antileishmanial drugs/agents

Quinazolinones, a class of nitrogen-containing heterocyclic compounds, are widely present in different natural products [17, 18]. They are known to exhibit diverse pharmacological activities including antimalarial [19, 20] and antileishmanial activities [21, 22]. For instance, febrifugine (**I**) and halofuginone (**II**), that possess the quinazolinone scaffold, shown to have potent antimalarial and antileishmanial activities [5] though their clinical use is hampered by poor safety profiles. Structure–activity relationship (SAR) studies on quinazolinone derivatives demonstrated that the presence of substituted phenyl moiety (2,3 or 4 methyl, methoxy, nitro, or a halogen) at the C-3 position of the quinazolinone moiety is beneficial for improved antimalarial activities [23]. Moreover, 2,3-disubstituted-4(3*H*)-quinazolinones with phenyl moieties at C-2 and a benzyl group at the C-3 position (**III**) also manifested promising in vitro antileishmanial activity *Leishmania major* with an IC_50_ value of 48.91 µg/mL (Fig. 1) [24]. Taking this into account, different efforts are being made to synthesize, 2,3-disubstituted-4(3*H*)-quinazolinones, especially 3-aryl-2-styryl substituted-4(3*H*)-quinazolinones in pursuit for potential antimalarial and antileishmanial agents. The lack of a versatile synthetic protocol is a bottleneck in the preparation of 3-aryl-2-styryl substituted-4(3*H*)-quinazolinone derivatives as only a few efficient synthetic methods are proved plausible and most of them tolerate only the attachment of phenyl groups at the C-2 position of the quinazolinone scaffold [25]. In this regard, the Knoevenagel condensation of 2-methyl substituted quinazolinones with aromatic aldehydes under acidic or basic [26, 27] conditions are the most frequently sought method for the preparation of 3-aryl-2-styryl substituted-4(3*H*)-quinazolinone derivatives. Thus, the aim of the present study was to synthesize some 3-aryl-2-styryl substituted-4(3*H*)-quinazolinone derivatives through the Knoevenagel condensation of 2-methyl-substituted quinazolinones and to assess their in vivo antimalarial activities, in vitro antileishmanial activities and in vivo oral acute toxicities in mice.

## Results and discussion

### Chemistry

**Fig. 2:**
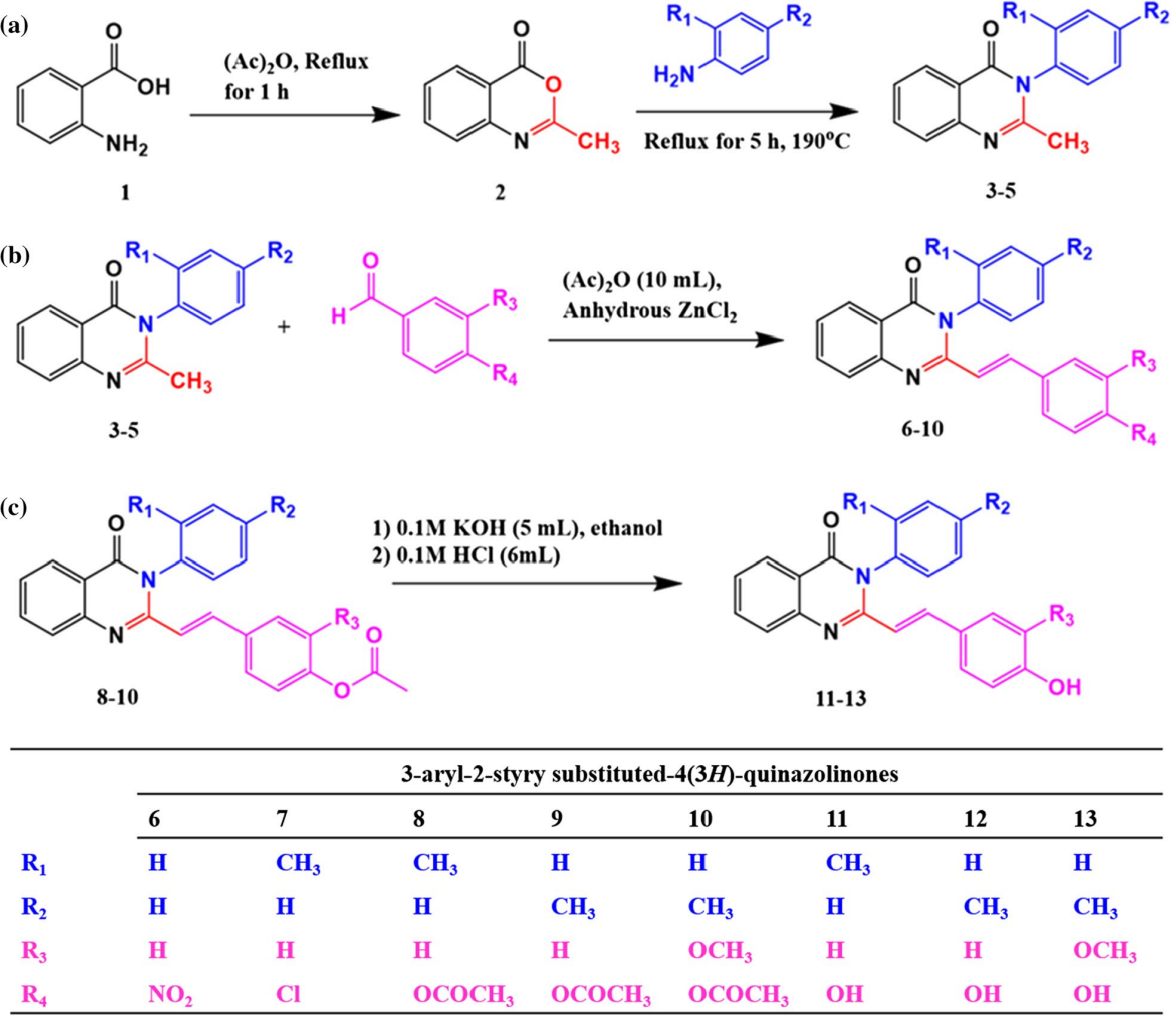
Schematic representation of the different reaction routes used in the synthesis of 3-aryl-2-substituted styryl-4(3*H*)-quinazolinones

Synthesis of 3-aryl-2-styryl substituted-4(3*H*)-quinazolinones (**6**–**13**) involved the formation of **2**–**5** as intermediates (Fig. 1). These compounds (**6**–**13**) were synthesized using the Knoevenagel condensation of 3-aryl-2-methyl substituted-4(3*H*)-quinazolinones (**3**–**5**) under acidic conditions. Though the Knoevenagel condensation suffers from certain drawbacks such as requiring multistep procedures, costly reagents, harsh reaction conditions, complex experimental processes, and low yields, it is synthetically useful as styryl substitution at C-2 is well tolerated [27]. In this study, the 3-aryl-2-styryl substituted-4(3*H*)-quinazolinones (**6**–**13**) were synthesized in good yields, which ranged from 58.4 to 86.5% which is comparable with the 2-styryl substituted-4(3*H*)-quinazolines prepared by one-pot tandem synthesis (70 to 82%) [28]. The chemical structures of the synthesized compounds were verified based on elemental microanalysis, IR, ^1^H NMR data (Additional files 1, 3, 4, 5, 6, 7, 8) and ^13^C NMR data (Additional file 2). For instance, the ^1^H NMR spectrum of compound **6** showed doublets at δ 7.36 and δ 7.46 for phenyl C_2_,_6_ and 4-nitrophenyl C_2_,_6_ protons respectively. The two protons of 4-nitrophenyl which are adjacent to the NO_2_ group are represented by a doublet at δ 8.18 (J = 8.80 Hz). Two doublets at δ 6.56 and 8.00 corresponding to styrene protons with a J value of 15.50 Hz, clearly indicated the transoidal attachment of hydrogens. All the ten aromatic protons appeared in the range of δ 6.65–8.35, thus confirming the successful synthesis of compound **6** (see the assigned structure of the compound in Fig. 2). Similarly, the structures of the other compounds were confirmed based on physical and spectral data. The distinctive stretching and bending IR vibration frequencies, the elemental microanalysis, and the ^1^H NMR chemical shift data for each of the synthesized compounds are summarized in the experimental section. Overall, all the target compounds were successfully synthesized having a yellow color (from light to deep yellow colors) and well-defined needle-shaped crystals which is a characteristic property of 2-styryl substituted 4(3*H*)-quinazolinone derivatives.

### Biological activity results

#### In vivo antimalarial activity results

In the present study, the in vivo antimalarial activities of the synthesized compounds were investigated using a mouse model which is ideal to elucidate the prodrug effects of the target compounds triggered by metabolic activation and eradication of infection due to the potential involvement of the immune system [29]. To achieve a steady state infection against the experimental animals, *Plasmodium berghei* ANKA strain served as a parasite in the experiment and the effects of the target compounds was assessed by the standard four-day suppressive test (often employed to evaluate early infections) [30] in terms of percent inhibition of parasitemia which is recognized as most trustworthy parameter in vivo antimalarial studies [31]. Herein, the standard antimalarial drug chloroquine phosphate (CQ) and equimolar amounts (48.46 µmol/Kg) of the target 3-aryl-2-styry substituted-4(3*H*)-quinazolinone derivatives were administered orally and the % suppression, parasitemia level, and the survival time of mice were compared against mice treated with a formulation containing a solution of 7% Tween 80, and 3% ethanol in distilled water (control group). As depicted in Table 1, the mean parasitemia level of mice treated with the target compounds decreased significantly (*p* ˂ 0.05) as compared to the mice in negative control group which confirmed the much anticipated antimalarial activities of the synthesized compounds in suppressing the proliferation and load of *Plasmodium berghei* ANKA strains. The significant antimalarial activities of the target compounds were also in agreement with that the activities of 4(3*H*)-quinazolinone derivatives on the same test strain [32]. The test compounds, **8**, **10**, **11**, **12**, and **13** exhibited a mean percent suppression of greater than 50%. On the other hand,** 6** and** 7** had less than 50% mean percent suppressions. Among the synthesized compounds **8** and **10** showed better antimalarial activities with percent suppression of 70.01 and 74.18 respectively. The mean parasitemia level of mice treated with **8** (19.57 ± 0.77) and **10** (16.85 ± 0.39) was found to be approximately four times lower than the negative control group (65.25 ± 0.73) showing the compounds have greatly reduced the parasite load. The better antimalarial activities of these compounds (**8** and **10**) are supported by a relative increment in mean survival time of 9.4 ± 0.37 and 9.8 ± 0.24, respectively [33, 34]. Hydrolysis (deacetylation) of compounds **8** and **10** resulted in the formation of compounds **11** and **13** which showed percent suppression of 61.27 and 63.02, respectively. These compounds displayed lower antimalarial activities as compared to compounds **8** and **10** with mean parasitemia levels of 25.27 ± 0.76 and 24.13 ± 0.29, respectively. The presence of acetyl groups at *para*-position compounds **8** and **10** might have played a potential role in their intestinal absorption and thereby improved antimalarial activities as compared to the deacetylated counterparts (compounds **11** and **13**). The result suggested that compounds **8** and **10** might elicit their antimalarial activities as prodrugs, which can be activated through cleavage of the acetyl moieties by enzymes once absorbed into the systemic circulation of test mice.

**Table 1:** Antimalarial activity test for the synthesized compounds at a dose of 48.46 µmol/kg

Test compounds	Structure*	Dose (mg/kg)	% Parasitemia	% Suppression	Mean survival time (day)
6		17.95	39.22 ± 0.7	38.89	6.5 ± 0.3
7		18.07	35.69 ± 0.69	45.3	6.9 ± 0.5
8		19.21	19.57 ± 0.77	70.01	9.4 ± 0.37
10		20.67	16.85 ± 0.39	74.18	9.8 ± 0.24
11		17.17	25.27 ± 0.76	61.27	7.3 ± 0.54
12		17.17	32 ± 0.5	50.96	7.6 ± 1.12
13		18.63	24.13 ± 0.29	63.02	7.9 ± 0.3
NC**	–	1 mL/100 g	65.25 ± 0.73	0.0	5.8 ± 0.17
CQ	–	25	0	100	ND

^*^The equimolar concentration of the synthesized compounds as compared to the reference drug, CQ

^**^Values are mean ± standard deviations, p ˂ 0.05

*NC* negative control, *CQ* chloroquine sulfate, ND, no mice death was recorded during the study period

#### In vitro antileishmanial activity results

The antipromastigote activities of the synthesized compounds and the existing antileishmanial drugs (miltefosine and amphotericin B deoxycholate) were assessed using the clinical isolate of the *Leishmania donovani* strain, which is known to cause VL worldwide. The soluble, stable and non-toxic fluorescence dye, AlamarBlue^®^ (resazurin) (Trek Diagnostic Systems, Inc., Cleveland, OH, USA) was used to quantitatively determine the viability of *Leishmania donovani* and compute the IC_50_ value of target compounds and the reference drugs [35]. Only metabolically active cells can reduce resazurin and convert into fluorescent resorufin. Thus, the fluorescent intensity of resorufin is directly correlated to the number of viable *Leishmania donovani* cells [36, 37]. In the present study, the in vitro results reiterated that compounds **6**, **10**, **11**, **12**, and **13** have displayed better inhibitory activities than the reference drug miltefosine (IC_50_ = 3.191 µg/mL) (Table 2). Compounds **7** and **8** (IC_50_ = 3.248 and 3.326 µg/mL respectively**)** were proved to be the least active as compared to the reference drug miltefosine. On the other hand, except for compound **6** (IC_50_ = 0.021 µg/mL), the synthesized compounds revealed lower inhibitory activities than the reference drug amphotericin B (IC_50_ = 0.046 µg/mL). Interestingly, (*E*)-2-(4-nitrostyryl)-3-phenylquinazolin-4(3*H*)-one (**6)** is two times and 150 times more active than the standard drugs amphotericin B and miltefosine respectively. The presence of strong interactions such as hydrogen bonding between compound **6** and amino acid residues in the active site of the enzyme that is responsible for survival and replication of *the Leishmania donovani* strain may be implicated in the superior antipromastigote activity of compound **6** [38]. Inspired by its promising antileishmanial outcome, (*E*)-2-(4-nitrostyryl)-3-phenylquinazolin-4(3*H*)-one (**6)** was subjected to molecular docking study by fitting it in Lm-PTR1 active site to gain an insight about the types of interactions between the simulated enzyme and itself.

**Table 2:** Data for antipromastigote activity (IC_50_) testing of the synthesized compounds

Test compounds	Structure	IC_50_ values (µg/mL)
6		0.021 ± 0.008
7		3.248 ± 0.282
8		3.326 ± 0.126
10		0.196 ± 0.042
11		0.185 ± 0.026
12		1.842 ± 0.168
13		2.265 ± 0.214
Miltefosine	–	3.191 ± 0.194
Amphotericin B	–	0.046 ± 0.009

#### Molecular docking study

**Fig. 3:**
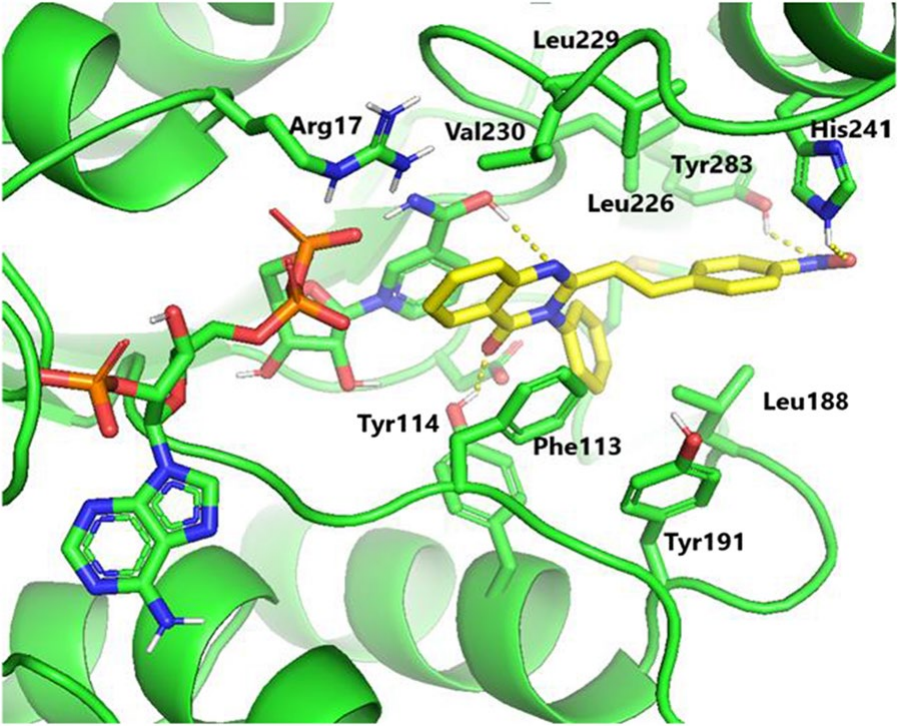
The docking pose of compound **6** as yellow sticks in the binding site of Lm-PTR1 (PDB code: 2bfm)

A molecular docking study was conducted to vindicate the observed in vitro antileishmanial activity of the most active compound **6**, where it is perfectly located in the binding site of Lm-PTR1 with binding free energy of − 9.2 kcal/mol. Insight analysis of the binding mode of compound **6** (Fig. 3), demonstrated H-bonding interactions with the catalytic residue of His241 as well as Tyr283 via its nitro functionally. Furthermore, the phenyl fragment of compound **6** appeared to be deeply packed between the side chains of Phe113, Leu188, Leu226, Leu229, and Val230 in the Lm-PTR1 active site. The existence of such types of hydrophobic interactions was beneficial for the promising in vitro antileishmanial activities of different compounds. The present molecular docking result is consistent with previous reports done on the same target, Lm-PTR1 [38, 39]. More interestingly, the quinazolinone scaffold exhibits H-bonding interactions as well as strong π-π stacking interactions with co-factor NADPH and Phe113. Moreover, the C = O group of quinazolinone formed H-bonding with Tyr114, in addition to other favorable polar interactions with Arg17, Asp181, and co-factor NADPH were mapped. These favorable hydrophilic and hydrophobic networks would stabilize compound **6** in the binding site of Lm-PTR1 and block the catalytic function of the leishmanial PTR1 enzyme, as well as justify its astonishing in vitro antileishmanial activity. In general, the in vitro antileishmanial activity and the molecular simulation results echoed the need for further detailed experiments to validate the broad-spectrum antileishmanial activity of compound **6** using different *Leishmania* strains and in vivo animal models.

#### Acute toxicity results

Dose-dependent oral acute toxicity study was conducted to assess the safety profiles of compounds **6**, **8** and **10** (which showed superior bioactivities) at 50, 100, 200, and 300 mg/kg. The result reiterated that none of the compounds caused mortality in mice at all dose levels within 24 h and the entire study period (14 days). In addition, there were no visible signs of overt toxicity in all mice subjected to physical and behavioral observations. Hence, it is fair to concluded that compounds **6, 8**, and **10** were devoid of any inherent toxicities at a tested dose of 300 mg/kg.

## Experimental

### Materials

#### Chemicals and reagents

Anthranilic acid, acetic anhydride, aniline, *p*-toluidine, *o*-toluidine, acetone, dimethylsulfoxide (DMSO), anhydrous zinc chloride, *p*-chlorobenzaldehyde, *p*-nitrobenzaldehyde, *p*-hydroxybenzaldehyde, chloroform, absolute ethanol, absolute methanol, resazurin sodium salt, anhydrous petroleum ether, distilled water, iodine, Giemsa stain, Tween 80, 1% acacia gum, HCl, and KOH were used in the study. Most of the commercial solvents, chemicals, and reagents were purchased from either Merck or Sigma-Aldrich with the highest purity and used without further purification.

#### Instruments and apparatuses

Melting points were determined in open capillaries using electro-thermal 9100 melting point apparatus and were uncorrected. The FTIR spectra in nujol were recorded with the SHIMADZU 8400SP FT-IR spectrophotometer (Shimadzu Corporation, Nakagyo-Ku, Kyoto, Japan), and nuclear magnetic resonance (NMR) spectral data were performed on Bruker Avance DMX400 FT-NMR spectrometer (Bruker, Billerica, MA, USA) using tetramethylsilane (TMS) as internal standard. Silica gel TLC plates of 0.25 mm thickness were used in the study.

#### Experimental animals and strains

Swiss albino male mice of weight 20–32 g and age 6–8 weeks were used for the antimalarial activity and acute toxicity tests. *Plasmodium berghei* ANKA strain is used to infect the mice for a four-day suppressive test, were obtained from Biomedical Laboratory, Department of Biology, Faculty of Science, AAU. *Leishmania donovani* isolate used in this study was obtained from the Leishmania Diagnosis and Research Laboratory (LDRL) culture bank, School of Medicine, AAU.

#### Culture medium and conditions

RPMI-1640, 10% heat-inactivated fetal calf serum (HIFCS), 1% penicillin–streptomycin, and 1% L-glutamine were supplied to make a complete culture medium. The *Leishmania donovani* isolate was grown first on Novy-MacNeal-Nicolle (NNN) medium and then in tissue-culture flasks containing RPMI 1640 medium supplemented with 10% HIFCS and 1% 100 IU penicillin/mL-100 µg/mL streptomycin solution at 22 ℃ for promastigotes [40].

#### Reference drugs

For the in vivo antimalarial activity testing, chloroquine phosphate (Ethiopian Pharmaceutical Manufacturer (EPHARM), Addis Ababa, Ethiopia) was used as a reference drug. Miltefosine/hexadecylphosphocholine (AG Scientific, San Diego, CA, USA) and amphotericin B deoxycholate (Fungizone^®^, ER Squibb, Middlesex, UK) were employed as reference drugs in the in vitro antileishmanial activity testing of the synthesized compounds.

### Methods

#### Synthesis of target compounds

The synthesis of 3-aryl-2-styryl substituted-4(*3H*)-quinazolinones, were achieved using 3-aryl-2-methyl-4(3*H*)-quinazolinones (**3**–**5**) as key intermediates. It involved cyclization, condensation, and hydrolysis reactions (Fig. 1). The details of each reaction and reaction conditions are mentioned in the following sections.

### General procedure for the synthesis of 2-methyl-3,1-benzoxazine-4-one

A solution of anthranilic acid (**1**) (10 g, 0.073 mol) in acetic anhydride (25 mL) was heated under reflux for 1 h. The precipitate formed on cooling was filtered and the excess acetic anhydride was washed with anhydrous petroleum ether, where a solid mass is obtained. The solid mass 2-methyl-3,1-benzoxazine-4-one (**2**), without purification, was used for the subsequent reaction [10, 40].

### General procedure for the synthesis of 3-aryl-2-methyl-4(3H)-quinazolinones

A mixture of 2-methyl-3,1-benzoxazine-4-one (**2**) (3 g, 0.017 mol) and equimolar amounts of aromatic amines (aniline, *o*-toluidine, and *p*-toluidine respectively) was heated under reflux at 190 ℃ for 5 h. The dark sticky mass formed was cooled and recrystallized from ethanol **(3–5)** [40].

### General procedure for the synthesis of 3-aryl-2-styryl substituted-4(3H)-quinazolinones

To a solution of each 3-aryl-2-methyl substrituted-4(3*H*)-quinazolinones (**3–5**) (0.5 g) in acetic anhydride (10 mL), an equimolar amount of the target aromatic aldehyde was added. Anhydrous zinc chloride (10 mg) is added as a catalyst. The reaction mixture is heated under reflux for 8 h, cooled, and poured into ice-cooled water. The solid products formed (**6–8** and **10)** were filtered, dried, and recrystallized from ethanol [26, 40].

### (E)-2-(4-nitrostyryl)-3-phenylquinazolin-4(3H)-one (6)

IR (Nujol) (cm^−1^): 1682 (C = O), 1593 (C = N), 1556 and 1377 (NO_2_). ^1^H NMR (CDCl_3_/CCl_4_) ppm: 6.56 (*d*, 1H, J = 15.5 Hz, vinyl-C_2_ H), 7.36 (*d*, 2H, J = 8.2 Hz, phenyl-C_2,6_ H), 7.46 (*d*, 2H, J = 8.7 Hz, 4-nitrophenyl C_2,6_ H), 7.52–7.56 (*m*, 1H, quina-C_6_ H), 7.60–7.66 (*m*, 3H, phenyl C_3,4,5_ H), 7.83–7.85 (*m*, 2H, quina-C_7,8_ H), 8.00 (*d*, J = 15.54 Hz, 1H, vinyl C_1_ H), 8.18 (*d*, 2H, J = 8.8 Hz, 4-nitrophenyl C_3,5_ H), 8.35 (*d*, J = 8.4 Hz, 1H, quina-C_5_ H). **Anal. calcd**. for C_22_H_16_N_3_O_3_: C, 71.34; H, 4.35; N, 11.35. **Found**: C, 71.21; H, 4.62; N, 11.58.

### (E)-2-(4-chlorostyryl)-3-o-tolylquinazolin-4(3H)-one (7)

IR (Nujol) (cm^−1^): 1682 (C = O), 1597 (C = N), 1224 (C–Cl). ^1^H NMR (CDCl_3_/CCl_4_) ppm: 2.15 (*s*, 3H, *o*-tolyl-CH_3_), 6.33 (*d*, 1H, J = 15.5 Hz, vinyl C_2_ H), 7.23–7.25 (*m*, 3H, 4-chlorophenyl C_3,5_ and *o*-tolyl C_3_ H), 7.30 (*d*, 2H, J = 8.6 Hz, 4-chlorophenyl C_2,6_ H), 7.41–7.54 (*m*, 4H, *o*-tolyl C_4,5,6_ and quina-C_6_ H), 7.84 (*m*, 2H, quina-C_7,8_ H), 7.99 (*d*, 1H, J = 15.5 Hz, vinyl C_1_ H), 8.36 (*d*, 1H, J = 7.9 Hz, quina-C_5_). **Anal. calcd**. for C_23_H_17_ClN_2_O: C, 73.69; H, 5.12; N, 7.47; Cl, 9.46. **Found**: C, 73.26; H, 4.89; N, 7.78; Cl, 9.67.

### 4-((1E)-2-(3,4-dihydro-4-oxo-3-o-tolylquinazolin-2-yl)vinyl)phenyl acetate (8)

IR (Nujol) (cm^−1^): 1756 (C = O), 1682 (C = O), 1634 (C = N), 1205 (C–O–C). ^1^H NMR (CDCl_3_/CCl_4_) ppm: 2.15 (*s*, 3H, phenylacetate-CH_3_), 2.35 (*s*, *3*H, *o*-tolyl-CH_3_), 6.31 (*d*, 1H, J = 15.5 Hz, vinyl C_2_ H), 7.06 (*d*, 2H, J = 8.6 Hz, phenylacetate C_3,5_ H), 7.25 (*d*, 1H, J = 7.7 Hz, *o*-tolyl C_3_ H), 7.34 (*d*, 2H, J = 8.6 Hz, phenylacetate C_2,6_ H), 7.41–7.51 (*m*, 4H, *o*-tolyl C_4,5,6_ and quina-C_6_ H), 7.823–7.832 (*m*, 2H, quina-C_7,8_ H), 8.02 (*d*, 1H, J = 15.5 Hz, vinyl C_1_ H), 8.35 (*d*, 1H, J = 7.9 Hz, quina-C_5_ H). **Anal. calcd**. for C_25_H_20_N_2_O_3_: C, 75.36; H, 5.57; N, 7.03. **Found**: C, 75.62; H, 5.32; N, 6.88.

### 4-((1E)-2-(3,4-dihydro-4-oxo-3-p-tolylquinazolin-2-yl)vinyl)-2-methoxyphenyl acetate (10)

IR (Nujol) (cm^−1^): 1761(C = O), 1682 (C = O), 1614 (C = N), 1260 and 1149 (C–O–C). ^1^H NMR (CDCl_3_/CCl_4_) ppm: 2.3 (*s*, 3H, phenylacetate CH_3_), 2.5 (*s*, 3H, *p*-tolyl-CH_3_), 3.8 (*s*, 3H, OCH_3_), 6.36 (*d*, 1H, J = 15.5 Hz, vinyl C_2_ H), 7.00 (*m*, 3H, 2-methoxyphenyl C_3,5,6_ H), 7.22 (*d*, 2H, J = 8.1 Hz, *p*-tolyl C_3,5_ H), 7.41 (*d*, 2H, J = 8.1 Hz, *p*-tolyl C_2,6_ H), 7.51 (*m*, 1H, quina-C_6_ H), 7.81 (*m*, 2H, quina-C_7,8_ H), 7.93 (*d*, 1H, J = 15.5 Hz, vinyl C_1_ H), 8.33 (*d*, 1H, J = 7.3 Hz, quina-C_5_ H). **Anal. calcd**. for C_26_H_22_N_2_O_4_: C, 72.88; H, 5.65; N, 6.54. **Found**: C, 73.12; H, 5.35; N, 6.32.

### General procedure for the synthesis of 3-aryl-2-(4-deacetylatedstyryl)-4(3H)-quinazolinones

Subsequent treatment of **8**–**10** with 0.1 M alcoholic KOH (5 mL) (KOH dissolved in ethanol) followed by 0.1 M HCl (6 mL) was done. The resulting 4-hydroxyl bearing target compounds (**11**–**13**) was precipitated, filtered, dried, and recrystallized from ethanol [26, 40].

### (E)-2-(4-hydroxystyryl)-3-o-tolylquinazolin-4(3H)-one (11)

IR (Nujol) (cm^−1^): 3274 (OH), 1651 (C = O) and 1606 (C = N). ^1^H NMR (DMSO-d_6_) ppm: 2.05 (*s*, 3H, *o*-tolyl-CH_3_), 6.05 (*d*, 1H, J = 15.4 Hz, vinyl C_2_ H), 6.75 (*d*, 2H, J = 8.6 Hz, 4-hydroxypheny C_3,5_ H), 7.20 (*d*, 2H, J = 8.6 Hz, 4-hydroxyphenyl C_2,6_ H), 7.38 (*d*, 1H, J = 7.5 Hz, *o*-tolyl C_3_ H), 7.43–7.44 (*m*, 1H, quina-C_6_ H), 7.50–7.54 (*m*, 3H, *o*-tolyl C_4,5,6_ H), 7.75–7.77 (*d*, 1H, J = 8.1 Hz, quina-C_8_ H), 7.85–7.89 (*m*, 2H, vinyl C_1_ and quina-C_7_ H), 8.14 (*d*, 1H, J = 9.49 Hz, quina-C_5_ H), 9.95 (*s*, 1H, phenolic-OH). **Anal. calcd**. for C_23_H_18_N_2_O_2_: C, 77.51; H, 5.66; N, 7.86. **Found**: C, 77.21; H, 5.78; N, 7.61.

### (E)-2-(4-hydroxystyryl)-3-p-tolylquinazolin-4(3H)-one (12)

IR (Nujol) (cm^−1^): 3296 (OH), 1651(C = O) and 1608 (C = N). ^1^H NMR (DMSO-d_6_) ppm: 2.44 (*s*, 3H, *p-*tolyl CH_3_), 6.16 (*d*, 1H, J = 15.4 Hz, vinyl C_2_ H), 6.76 (*d*, 2H, J = 8.4 Hz, 4-hydroxyphenyl C_3,5_ H), 7.22 (*d*, 2H, J = 8.4 Hz, 4-hydroxyphenyl C_2.6_ H), 7.32 (*d*, 2H, J = 8.06 Hz, *p-*tolyl C_3,5_ H), 7.42 (*d*, 2H, J = 7.9 Hz, *p*-tolyl C_2,6_ H), 7.476–7.513 (*m*, 1H, quina-C_6_ H), 7.746 (*d*, 1H, J = 8.2 Hz, quina-C_8_), 7.78–7.86 (*m*, 2H, quina-C_7_ and vinyl C_1_ H), 8.11 (*d*, 1H, J = 8.0 Hz, quina-C_5_ H), 9.98 (*s*, 1H, phenolic-OH). **Anal. calcd**. for C_23_H_18_N_2_O_2_: C, 77.51; H, 5.66; N, 7.86. **Found**: C, 77.21; H, 5.26; N, 7.48.

### (E)-2-(4-hydroxy-3-methoxystyryl)-3-p-tolylquinazolin-4(3H)-one (13)

IR (Nujol) (cm^−1^): 3423 (OH), 1683 (C = O), 1634 (C = N), 1211and 1148 (C–O–C). ^1^H NMR (CDCl_3_/CCl_4_) ppm: 2.5 (*s*, 3H, *p-*tolyl CH_3_), 3.8 (*s*, 3H, OCH_3_), 5.95 (*s*, 1H, phenolic-OH), 6.3 (*d*, 1H, J = 15.4 Hz, vinyl C_2_ H), 6.82–6.92 (*m*, 3H, 4-hydroxy-3-methoxyphenyl C_3,5,6_ H), 7.22 (*d*, 2H, J = 8.3 Hz, *p*-tolyl C_3,5_ H), 7.40 (*d*, 2H, J = 8.0 Hz, *p*-tolyl C_2,6_ H), 7.48 (*m*,1H, quina-C_6_ H), 7.78–7.79 (*m*, 2H, quina-C_7,8_ H), 7.92 (*d*, 1H, J = 15.4 Hz, vinyl C_2_ H), 8.30–8.32 (*d*, 1H, J = 7.9 Hz, quina-C_5_ H). **Anal. calcd**. for C_24_H_20_N_2_O_3_: C, 74.59, H, 5.74; N, 7.23. **Found**: C, 74.43; H, 5.48; N, 6.92.

#### Preparation of stock and working solutions

Stock solutions of 10 mg/mL of the synthesized compounds were prepared by dissolving each compound in DMSO for the antileishmanial activity testing. Stock solutions were diluted using complete RPMI to obtain aliquots of 10 µg/mL. Then, three-fold serial dilution with complete RPMI gave the final six working concentrations (10, 3.33, 1.11, 0.37, 0.12, and 0.04 µg/mL) of each of the synthesized compounds. Amphotericin B deoxycholate and miltefosine, which were used as positive controls for the comparison of the antileishmanial activities of the test compounds, were also made in three-fold serial dilutions. All the prepared drugs were stored at − 20 °C and retrieved only during use [41].

#### In vivo* a*ntimalarial activity test

The antimalarial activities of the target compounds were assessed by the four-day standard suppressive test against mice infected with *Plasmodium berghei* [42, 43]. The mice were acclimatized to the experimental conditions in the animal house of Biomedical Laboratory, Addis Ababa University, Faculty of Science, Department of Biology, for seven days prior to the in vivo antimalarial activity testing. In due course, the mice were subjected to live in standard cages with a pelleted diet and water. To trigger a steadily rising infection in mice, blood infected with *Plasmodium berghei* ANKA strain (approximately 20–30% level of parasitemia) was collected from a donor mice using a syringe supplemented with 0.5% trisodium citrate and diluted in physiological saline to 10^7^ parasitized erythrocytes per mL. Then, 0.2 mL of inoculum (supposed to have about 2 × 10^7^ parasites) was injected into each mouse through the intraperitoneal route to achieve the desired level of infection within a short period of time [44]. After 2 h of post-infection, the mice were carefully weighed using a digital balance and randomly assigned into nine groups where each group contain five mice. Of which, mice assigned as a negative control (Group **1**) subjected to the formulation composed of 7% Tween 80, and 3% ethanol in distilled water (2 mL/100 g). In addition, mice which belong to the positive control (Group **2**) was treated with 25 mg/kg/day (0.04846 mmol/kg/day) of chloroquine phosphate (CQ). The remaining groups (**3–9**) were subjected to oral treatment with equimolar amounts of the respective target compounds for four consecutive days. Finally, a blood smear was taken from the tail of the mice in the 5th day (24 h after the last dose), air-dried, fixed with absolute methanol and treated with 6% Giemsa stain. The mean parasitemia level of each group (control and treatment groups) was computed microscopically by counting the number of erythrocytes in four fields (⁓100 erythrocytes per field). In the end, the antimalarial activities of the target compounds were expressed in terms of blood parasitemia, percent suppression and mean survival time of mice in comparison with the negative- and positive-control groups [45].

#### In vitro antileishmanial activity test

Prior to the in vitro antipromastigote testing, serial concentrations of the target compounds were prepared by implementing three-fold dilutions. Then, in a 96-well microtiter plate, 100 µl of each of the three-fold serial dilutions of the target compounds were added in triplicate wells. Then 100 µl of *Leishmania donovani* suspension containing 3.0 × 10^6^ promastigotes per milliliter were added in duplicates. In this experiment, the media and DMSO served as a negative control. Moreover, the concentration of DMSO was maintained below 1% of the target compound preparation to avoid the potential growth inhibition and interference effect of DMSO in the entire antipromastigote assay [40]. Properly labelled plates which belong to the negative-control group, positive-control groups (treated with miltefosine and amphotericin B deoxycholate) and target compounds were then kept in a humidified atmosphere at 22 ℃ under 5% CO_2_ for 68. To determine the viability *Leishmania donovani* isolates after the respective treatment, 10 µL of fluorochrome AlamarBlue^®^ solution prepared by dissolving 12.5 mg resazurin in 100 mL of distilled water was added to each well and incubated for 4 h. Then, the fluorescence intensity of each well was determined using Victor3 Multilabel Counter (PerkinElmer, Waltham, MA, USA), at 530 nm and 590 nm, excitation and emission wavelengths, respectively [46]. The IC_50_ values of the negative-control, positive-control groups and test-groups were determined from sigmoidal dose-response curves using GraphPad Prism 5.0 software (GraphPad Software, Inc., San Diego, CA, USA).

#### Molecular docking study

Molecular docking study was carried out for compound **6** which elicited pronounced antileishmanial activity, using AutoDock Vina by retrieving the three-dimensional structure of Lm-PTR1 from the Protein Data Bank (PDB ID: 2bfm). The complex with Trimethoprim forming by chain A of the Lm-PTR1 heterodimer was employed in the modeling study. Ligands were sketched in a manner which minimized energy and the protein was formulated via the Discovery studio suite (V5.1). The Python script (prepare receptor4.py) offered by the MGLTools package (version 1.5.4) was followed to convert protein files to PDBQT format for docking using AutoDock Vina (version 1.1.2). The efficiency of the search algorithm was maintained at its default setting. The grid box docking dimensions were -3.629 Å × 41.782 Å × 66.219 Å, with a spacing of 1 Å to deal with all the possible conformations of the docked molecule. All graphical representations in Fig. 3 were extracted using Pymol.

#### In vivo* a*cute toxicity test

The preliminary safety profiles of target compounds (**6**, **8**, and **10**) with pronounced bioactivities were investigated using male Swiss albino mice (⁓20 g each) [43]. For this purpose, the mice were randomly allocated into six groups each containing six mice. The first five groups of mice (Group **1–5**) received each target compound solubilized using 1% gum acacia at a dose of 10, 50, 100, 200 and 300 mg/kg, respectively. The mice in last group (group **6**, served as a negative control) were orally treated with the solvent used to dissolve the target compounds (1% gum acacia) at a maximum dose of 1 mL/100 gm of body weight [47]. Each mouse was then monitored for gross changes such as salivation, loss of appetite, lacrimation, hair erection, diarrhea, convulsions, death, and other signs of overt toxicity.

#### Ethical consideration

The protocols that involving experimental animals were assessed and approved by the Institutional Ethics Review Committee, School of Pharmacy, Addis Ababa University. In addition, the study reported in accordance with the Animal Research Reporting of in vivo Experiments (ARRIVE) guidelines [48] and were handled according to the Guide for the Care and Use of Laboratory Animals (https://olaw.nih.gov/sites/default/files/Guide-for-the-Care-and-Use-of-Laboratory-Animals.pdf).

#### Statistical analysis

The antimalarial activities of the synthesized compounds were computed as mean ± standard deviation and one-way ANOVA was used to test the statistical significance for the suppressive test using Origin 6.0 software. Data on % suppression, % parasitemia and mean survival time was analyzed using Microsoft office excel 2007. Moreover, all the data were analyzed at a 95% confidence interval. The IC_50_ values for in vitro promastigotes assay of target compounds were determined from sigmoidal dose-response curves using computer software GraphPad Prism 5.0.

## Conclusion

Some novel 3-aryl-2-styryl substituted-4(3*H*)-quinazolinone were synthesized and tested for their antimalarial and antileishmanial activities. Compounds **8** and **10** showed pronounced antimalarial activities with percent suppression of 70.01 and 74.18 respectively. In addition, all the target compounds exhibited comparable or better antileishmanial effects as compared to the conventional drug, miltefosine. The compound (*E*)-2-(4-nitrostyryl)-3-phenylquinazolin-4(3*H*)-one (**6**) showed two times and 150 times better antileishmanial activities than miltefosine and amphotericin B. Moreover, the molecular simulation study revealed the presence of favorable interaction between compound **6** and the Lm-PTR1 active site, justifying its strong in vitro antileishmanial activity. Overall 3-aryl-2-styryl substituted-4(3*H*)-quinazolinone derivatives may represent an appealing class of heterocyclic compounds for the development of a new class of antimalarial and antileishmanial agents.

## Supplementary Information

**Additional file 1. Figure S1** ^1^H NMR spectrum of compound 6 in CDCl_3_.

**Additional file 2. Figure S2** ^13^C NMR spectrum of compound 6 in CDCl_3_.

**Additional file 3. Figure S3** ^1^H NMR spectrum of compound 7 in CDCl_3_.

**Additional file 4. Figure S4** ^1^H NMR spectrum of compound 8 in CDCl_3_.

**Additional file 5. Figure S5** ^1^H NMR spectrum of compound 10 in CDCl_3_.

**Additional file 6. Figure S6** ^1^H NMR spectrum of compound 11 in DMSO-d_6_.

**Additional file 7. Figure S7** ^1^H NMR spectrum of compound 12 in DMSO-d_6_.

**Additional file 8. Figure S8** ^1^H NMR spectrum of compound 13 in CDCl_3_.

## Data Availability

The datasets supporting the findings of this article are presented in the main manuscript. The ^1^H NMR and IR spectra of the synthesized compounds can be shared from the corresponding author on reasonable request.
