# Supplementary figures and images for: Synthesis, antimalarial, antileishmanial evaluation, and molecular docking study of some 3-aryl-2-styryl substituted-4(3H)-quinazolinone derivatives
Source: BMC Chem. 2022 Dec 2;16(1):107. doi: 10.1186/s13065-022-00903-0 (PMC9716151; doi:10.1186/s13065-022-00903-0)

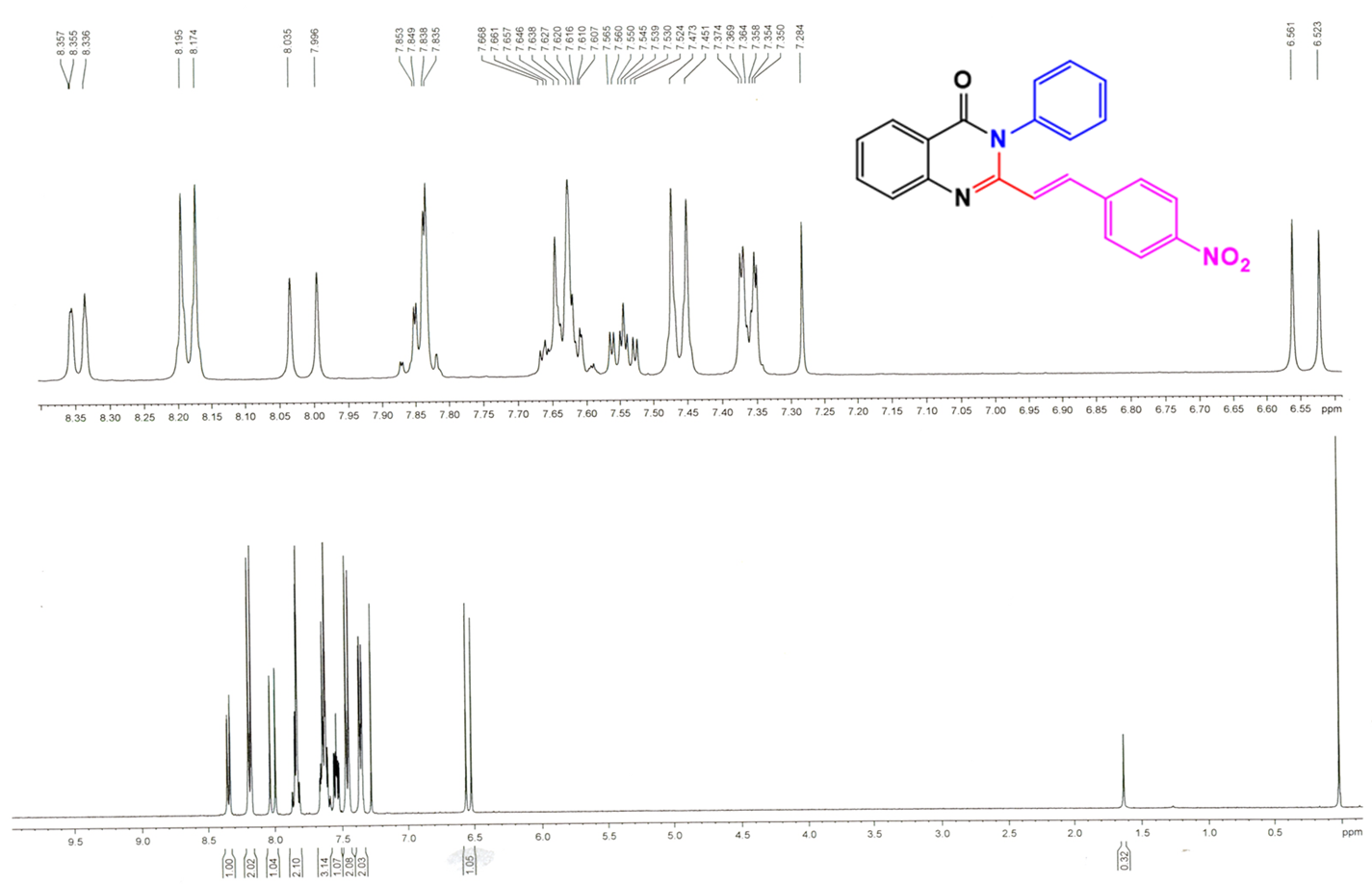


**Figure S1**: 1H NMR spectrum of compound **6** inCDCl3

Supplement: Supplementary file 1 — Additional file 1. Figure S1 1H NMR spectrum of compound 6 in CDCl3. [file 13065_2022_903_MOESM1_ESM.docx]

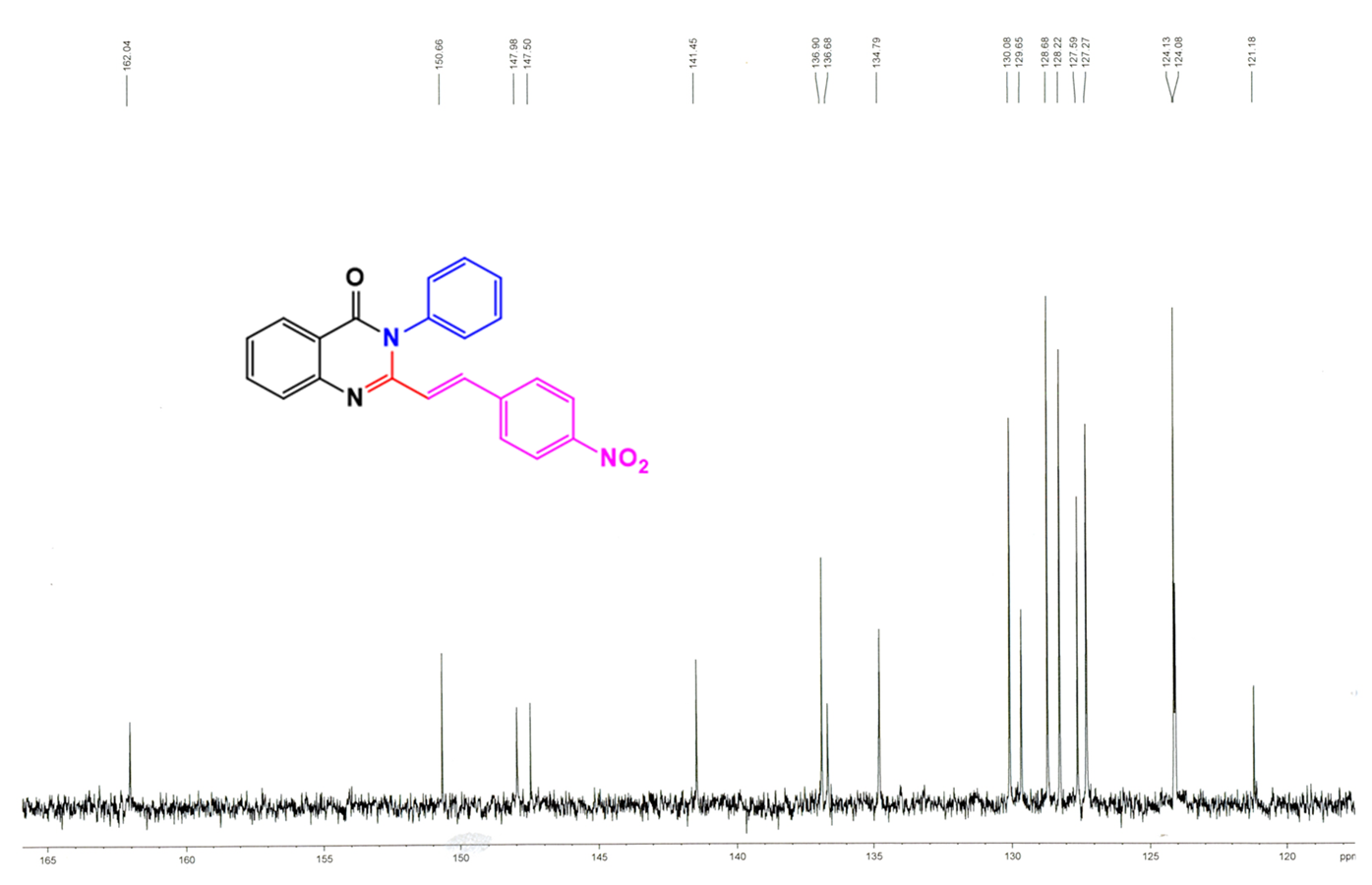


**Figure S2**: 13C NMR spectrum of compound **6** inCDCl3

Supplement: Supplementary file 2 — Additional file 2. Figure S2 13C NMR spectrum of compound 6 in CDCl3. [file 13065_2022_903_MOESM2_ESM.docx]

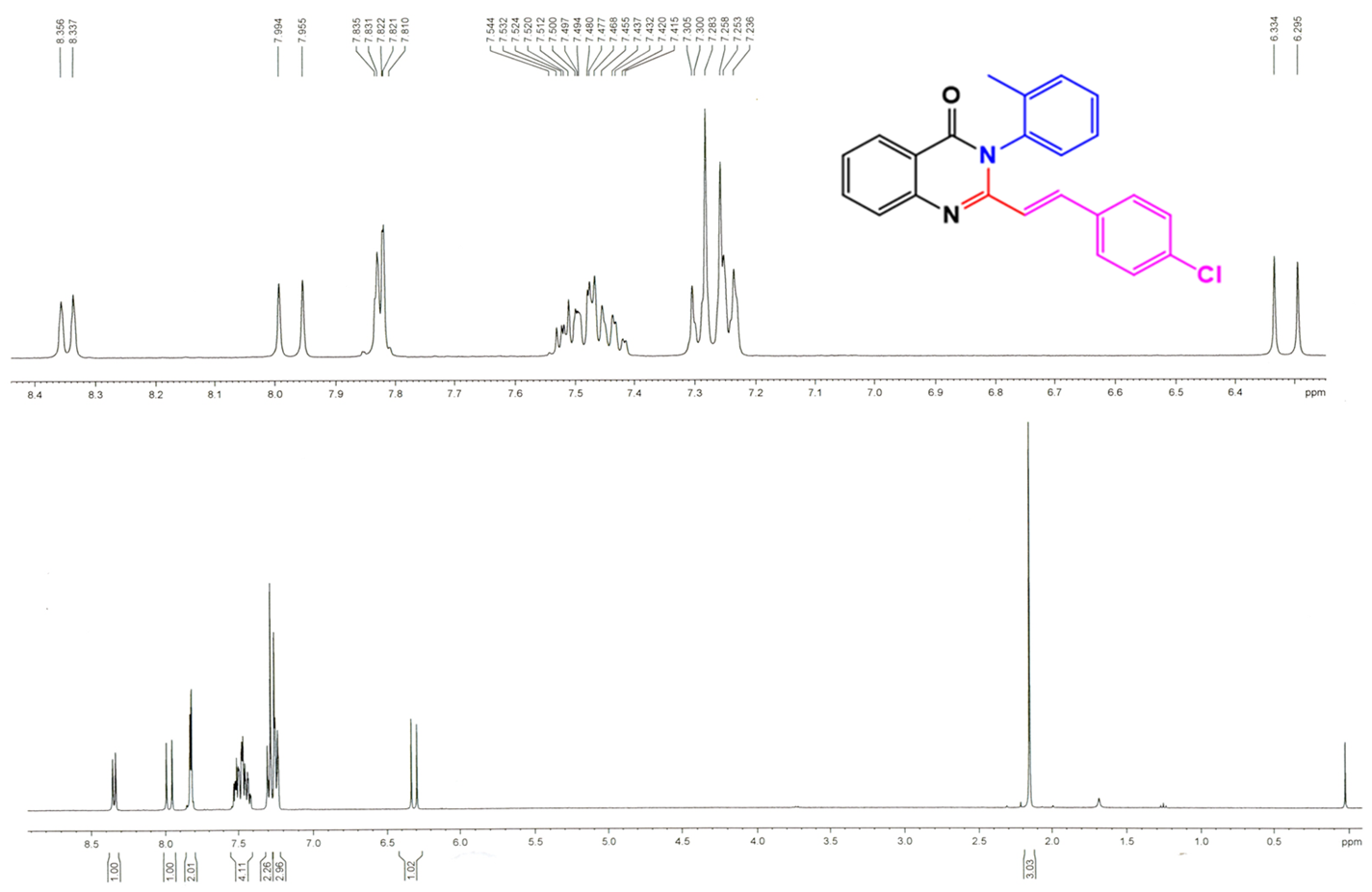


**Figure S3**: 1H NMR spectrum of compound **7** inCDCl3

Supplement: Supplementary file 3 — Additional file 3. Figure S3 1H NMR spectrum of compound 7 in CDCl3. [file 13065_2022_903_MOESM3_ESM.docx]

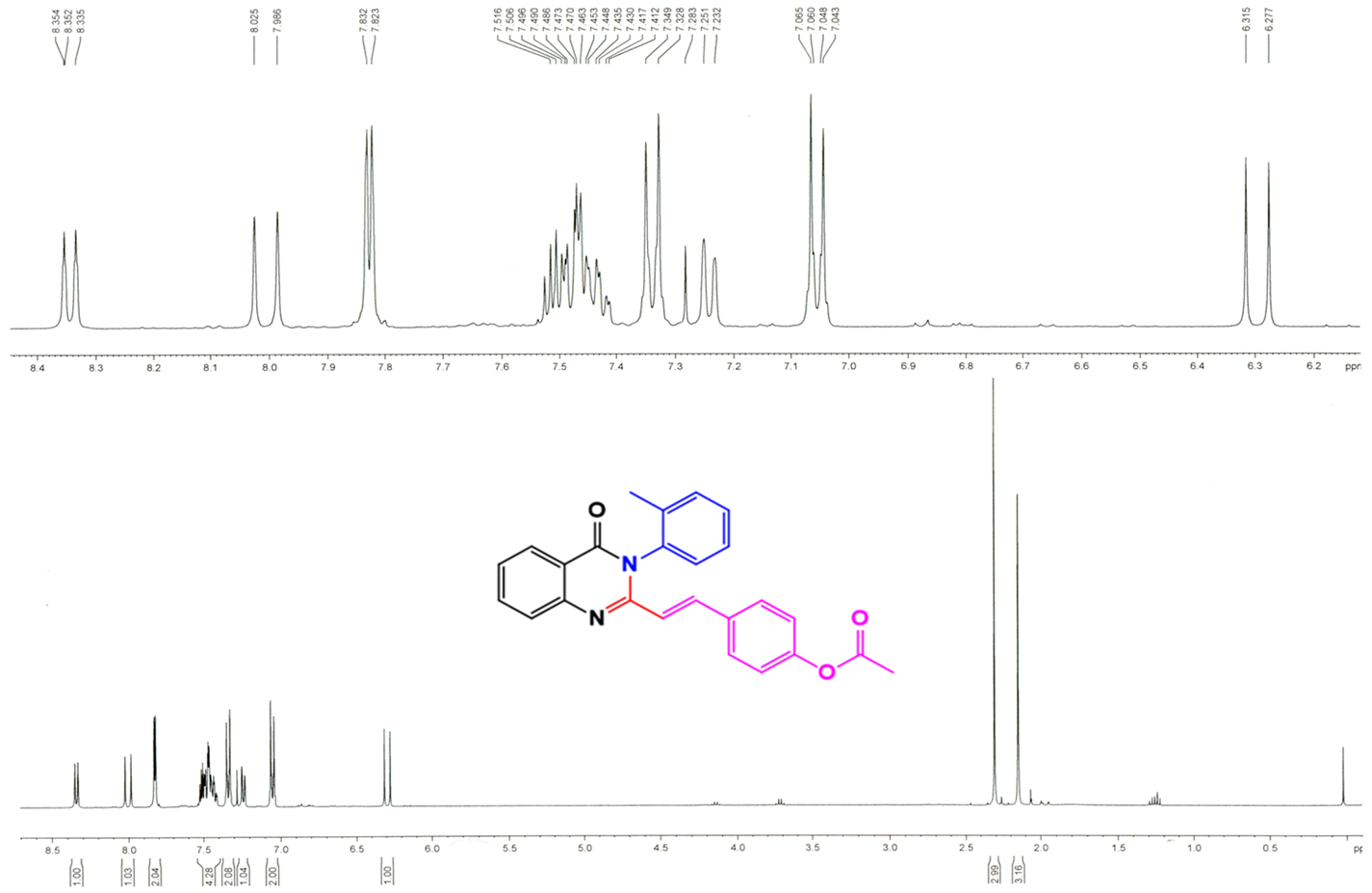


**Figure S4**: 1H NMR spectrum of compound **8** inCDCl3

Supplement: Supplementary file 4 — Additional file 4. Figure S4 1H NMR spectrum of compound 8 in CDCl3. [file 13065_2022_903_MOESM4_ESM.docx]

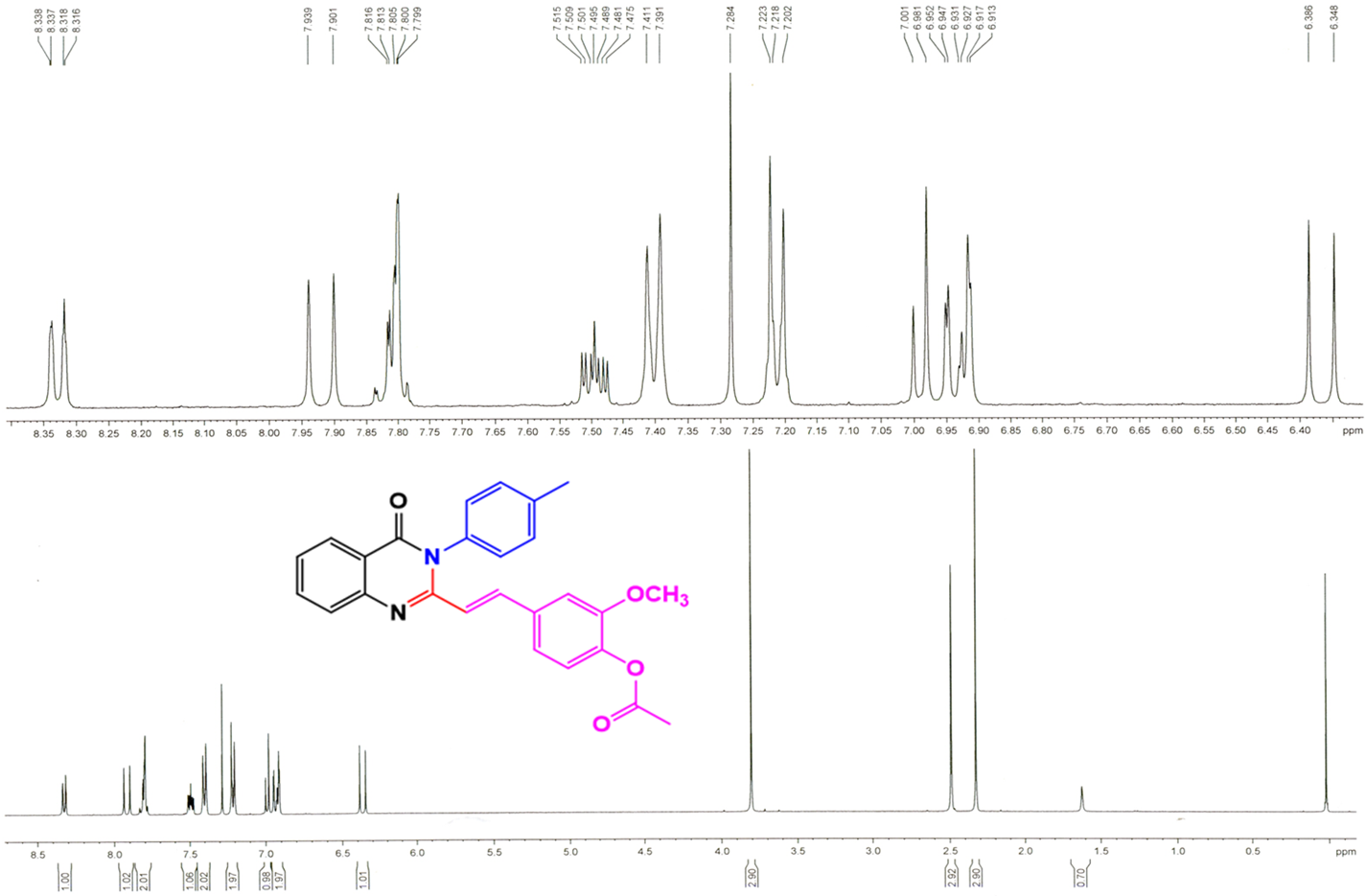


**Figure S5**: 1H NMR spectrum of compound **10** inCDCl3

Supplement: Supplementary file 5 — Additional file 5. Figure S5 1H NMR spectrum of compound 10 in CDCl3. [file 13065_2022_903_MOESM5_ESM.docx]

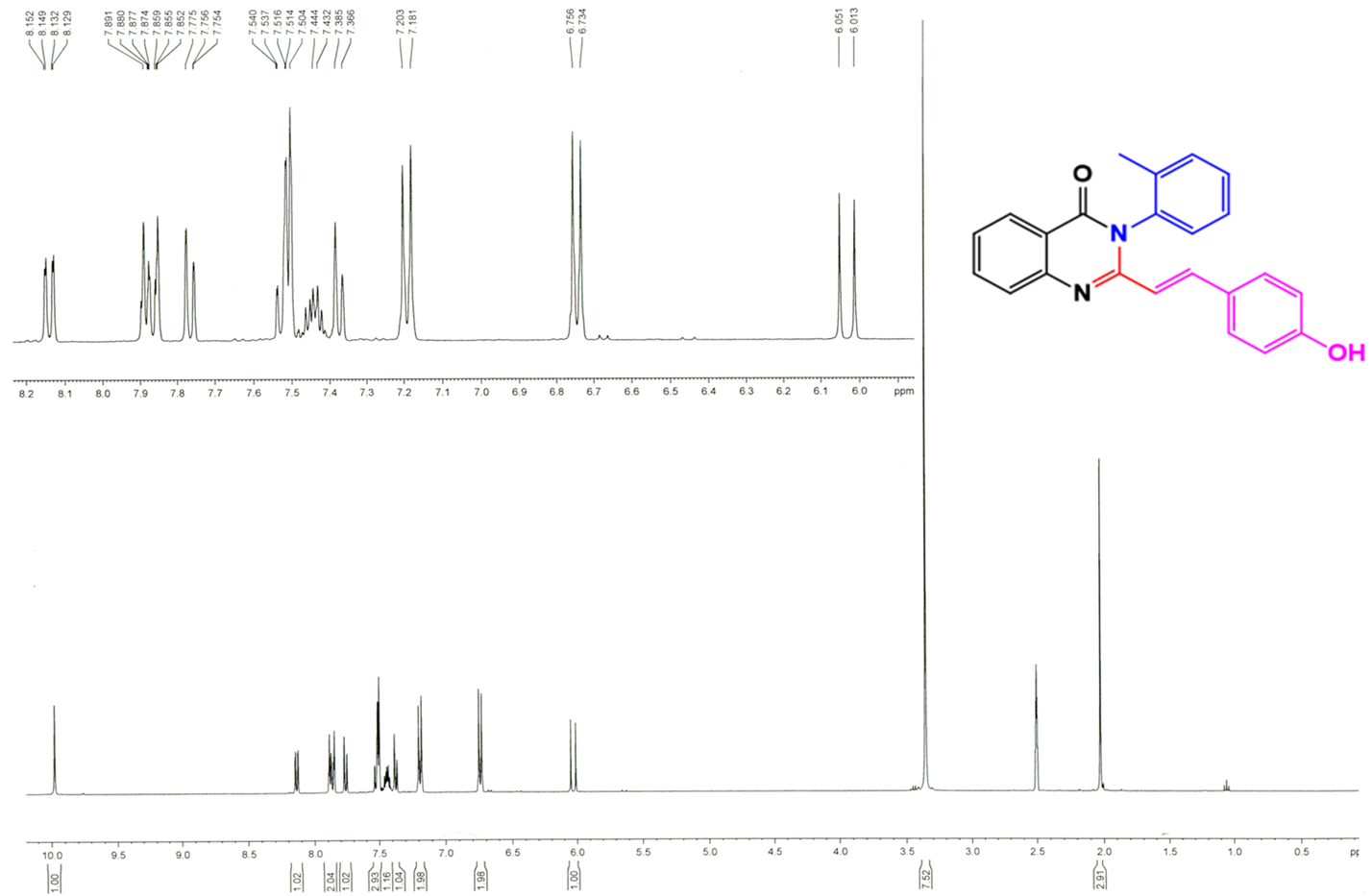


**Figure S6**: 1H NMR spectrum of compound **11** inDMSO-d6

Supplement: Supplementary file 6 — Additional file 6. Figure S6 1H NMR spectrum of compound 11 in DMSO-d6. [file 13065_2022_903_MOESM6_ESM.docx]

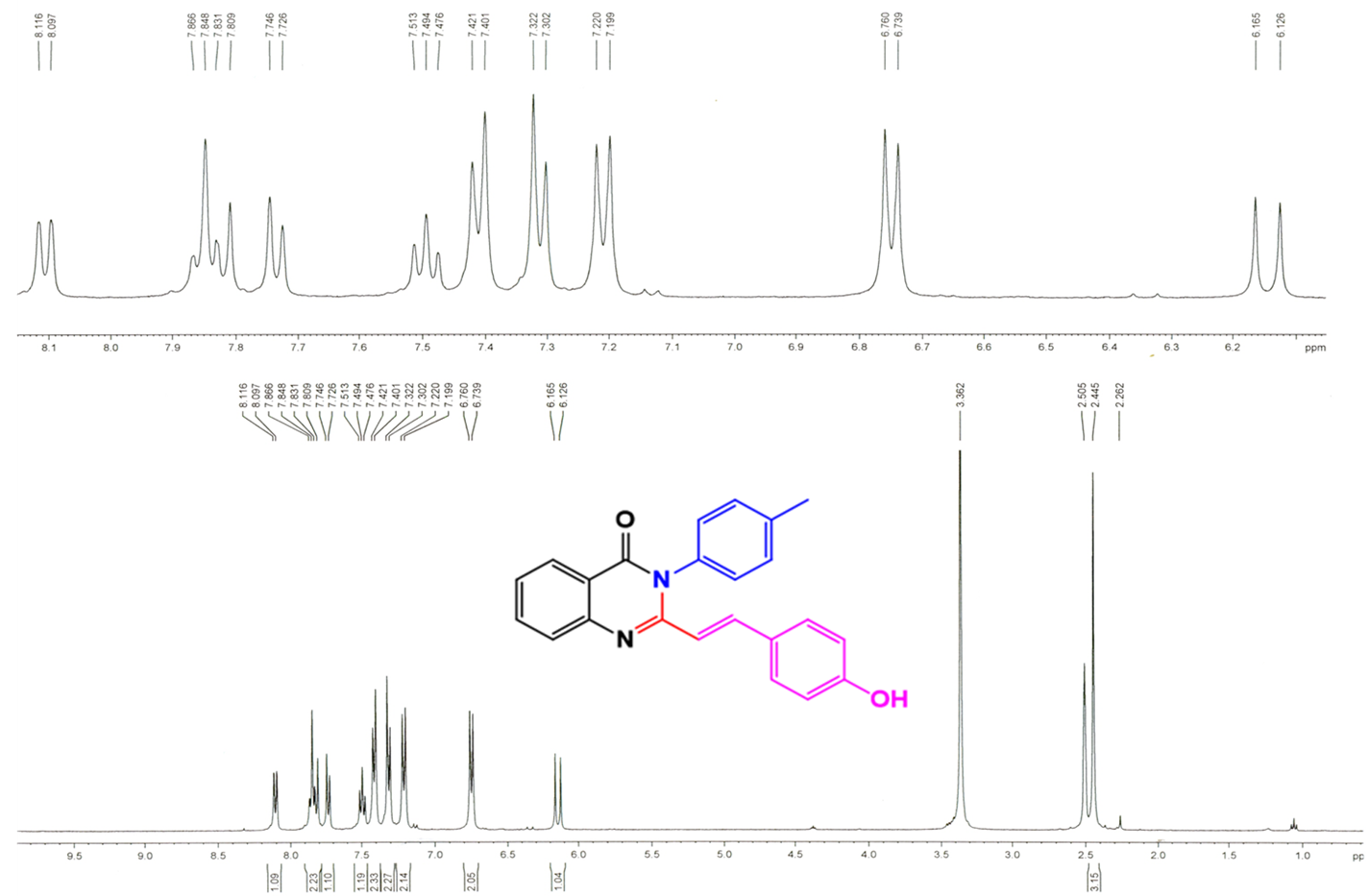


**Figure S7**: 1H NMR spectrum of compound **12** inDMSO-d6

Supplement: Supplementary file 7 — Additional file 7. Figure S7 1H NMR spectrum of compound 12 in DMSO-d6. [file 13065_2022_903_MOESM7_ESM.docx]

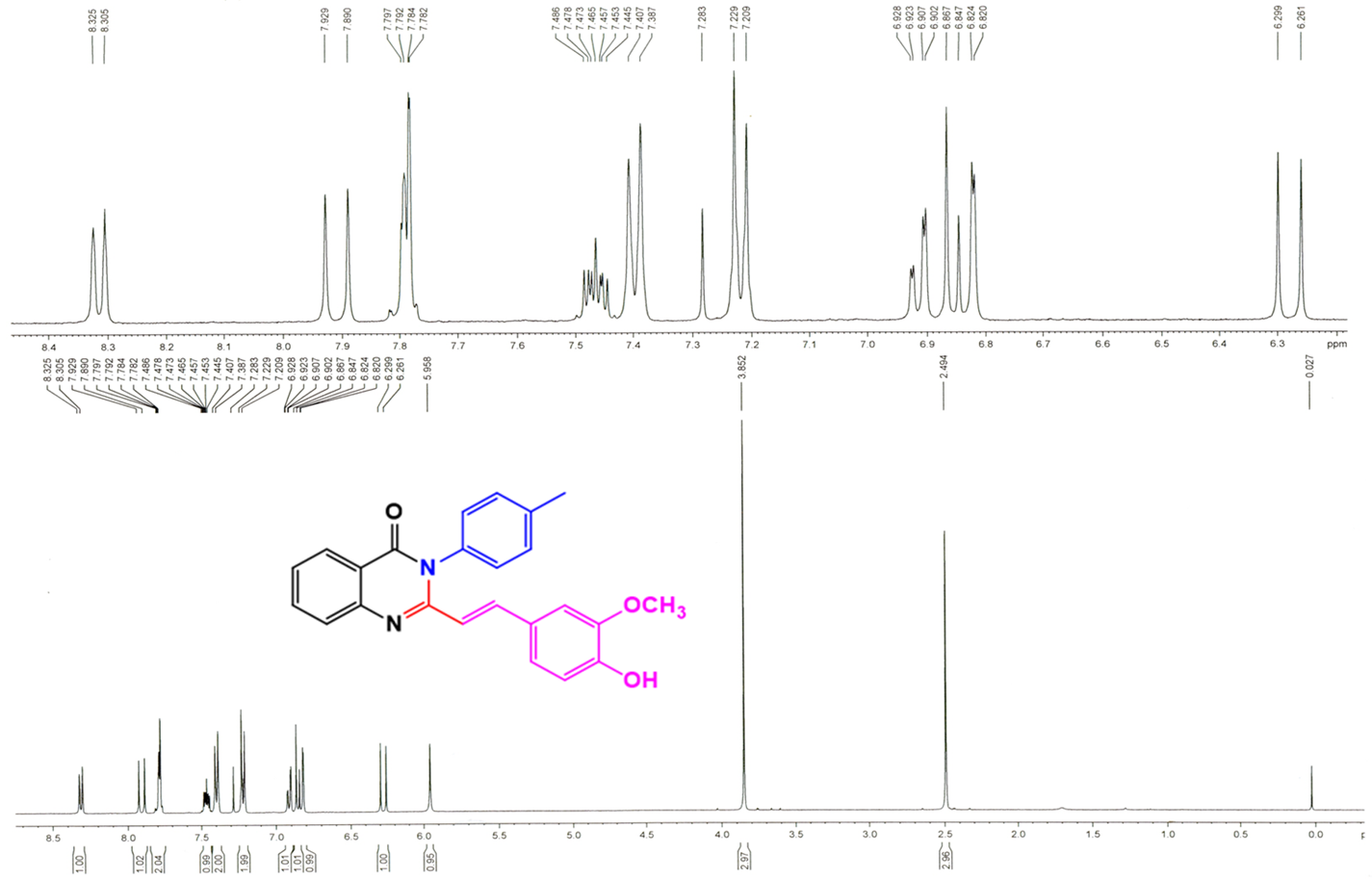


**Figure S8**: 1H NMR spectrum of compound **13** inCDCl3

Supplement: Supplementary file 8 — Additional file 8. Figure S8 1H NMR spectrum of compound 13 in CDCl3. [file 13065_2022_903_MOESM8_ESM.docx]
